# Tuning the Anti(myco)bacterial Activity of 3-Hydroxy-4-pyridinone Chelators through Fluorophores

**DOI:** 10.3390/ph11040110

**Published:** 2018-10-20

**Authors:** Maria Rangel, Tânia Moniz, André M. N. Silva, Andreia Leite

**Affiliations:** 1 REQUIMTE-LAQV, Instituto de Ciências Biomédicas de Abel Salazar, Universidade do Porto, 4050-313 Porto, Portugal; 2 REQUIMTE-LAQV, Departamento de Química e Bioquímica, Faculdade de Ciências, Universidade do Porto, 40169-007 Porto, Portugal; tania.moniz@fc.up.pt (T.M.); andre.silva@fc.up.pt (A.M.N.S.); acleite@fc.up.pt (A.L.)

**Keywords:** fluorescent iron chelator, 3-hydroxy-4-pyridinone, fluorophore, rhodamine, membrane interactions, bacteria, antibacterial activity

## Abstract

Controlling the sources of Fe available to pathogens is one of the possible strategies that can be successfully used by novel antibacterial drugs. We focused our interest on the design of chelators to address *Mycobacterium avium* infections. Taking into account the molecular structure of mycobacterial siderophores and considering that new chelators must be able to compete for Fe(III), we selected ligands of the 3-hydroxy-4-pyridinone class to achieve our purpose. After choosing the type of chelating unit it was also our objective to design chelators that could be monitored inside the cell and for that reason we designed chelators that could be functionalized with fluorophores. We didn’t realize at the time that the incorporation a fluorophore, to allow spectroscopic detection, would be so relevant for the antimycobacterial effect or to determine the affinity of the chelators towards biological membranes. From a biophysical perspective, this is a fascinating illustration of the fact that functionalization of a molecule with a particular label may lead to a change in its membrane permeation properties and result in a dramatic change in biological activity. For that reason we believe it is interesting to give a critical account of our entire work in this area and justify the statement “to label means to change”. New perspectives regarding combined therapeutic approaches and the use of rhodamine B conjugates to target closely related problems such as bacterial resistance and biofilm production are also discussed.

## 1. Introduction

### 1.1. Chelators and Iron

Numerous transition metal ions have proved to be essential for life, though it is well known that transition metals can be toxic. Living organisms have resolved this paradigm by incorporating metal ions in complex biological structures and by developing highly complex regulatory mechanisms to keep the amounts of “free metal ion” available at any time very tiny, thus counteracting toxicity [1].

Chelation is a type of bonding normally used to bind metal ions to biomolecules, namely proteins and nucleic acids, as well as to uptake and/or deliver metal ions. A chelator is a ligand (molecule or anion; Lewis base) that has the capacity to bind a metal ion (Lewis acid) through at least two coordinate bonds. The chemical entity that is formed upon chelation is designated a metal-chelate. There is a large variety of molecules that can act as chelators and which use for chelation: (a) different types of atoms (O, N, S) providing different coordination spheres and (b) different number of atoms (2, 3, 4, 6, etc.) being classified as having different denticity (bidentate, tridentate, tetradentate, hexadentate, etc.).

The affinity of a chelator for a selected metal ion, in a particular oxidation state, is defined by the stability constant (*Log β*) of the respective metal-chelate measured in standard thermodynamic conditions. The enhancement of stability, provided by replacement of ligands of lower denticity by chelators of higher denticity is known as the chelate effect [1].

It is important to bear in mind that, upon chelation the redox potential of the metal ion and protonation constants of the chelator are altered. In the case of iron (Fe), the first characteristic is particularly important since it allows the fine-tuning of redox potentials by choosing the appropriate ligands thus making it suitable to participate in electron transfer chains and providing an approach to diminish toxicity.

Iron is one of the most abundant elements in Earth’s crust, and the most abundant transition metal, being an essential micronutrient for all living organisms [2], with the exceptions of *Lactobacilli* and *Borrelia burgorferi* [3,4,5]. Despite its abundance, the geological availability of Fe is compromised by the fact that the element exists in insoluble chemical forms making its uptake extremely difficult to living organisms. Bacteria and plants obtain iron from the environment by chelation, whereby the element is chemically bound to another substance (siderophore) making the whole complex (Fe-chelate) soluble and available.

Siderophores are chelators produced by living organisms to acquire Fe from the environment in response to a need of the element. More than 500 siderophores have been described and the chemical structure of approximately 260 siderophores is known [6]. Most siderophores are hexadentate chelators bearing hydroxamate (such as desferrioxamine) or catecholate units (such as enterobactin) for which the values of the Fe(III) stability constants are in the range 30 < (*Log β*) < 49. The latter values are *ca* 20 orders of magnitude higher than those regarding the corresponding Fe(II) chelates thus favouring the release of the metal ion by reduction, a common step in the delivery of Fe into cells [7]. At this point it must be mentioned that, in physiological conditions, judgement of the effectiveness of a chelator to withdraw Fe(III) from the environment is better achieved by comparison of the respective pFe(III) (-log[Fe(III)]) values, which are calculated considering pH 7.4, a total ligand concentration of 10^−5^ M and a total Fe(III) concentration of 10^−6^ M [8] in opposition to the standard thermodynamic conditions considered for the determination of the stability constant (*Log β*) [9].

Iron is a cofactor in metalloproteins, and is involved in essential biological functions, from DNA biosynthesis and transcription to energy production and central metabolism [10]. The suitability of Fe arises from its unique physico-chemical properties to act in electron transfer, namely the possibility of existing in various oxidation states and the extreme variability of its redox potentials, which can be fine-tuned upon chelation [11,12]. Nevertheless, this redox activity can also be pernicious if the element exists in chemical forms with redox potentials that allow their participation in the generation of reactive oxygen species (ROS) [13].

The maintenance of Fe homeostasis demands a tight control of intracellular and systemic iron levels, which is achieved through sophisticated regulatory mechanisms [14]. Disruption of Fe homeostasis has consequences in the physiopathology and clinical evolution of a significant number of diseases in all medical areas. In clinical practice, and depending on the disease, various therapeutic approaches are used to regain Fe homeostasis and chelation therapy is one of the currently available choices [15,16,17,18,19].

The need to control Fe levels by chelation, in Fe unbalance related diseases, has prompted chemists to synthesize several types of chelators and the increasing knowledge about Fe metabolism and identification of specific targets, has been providing important feedback to the design of new and more effective chelators.

**Figure 1:**
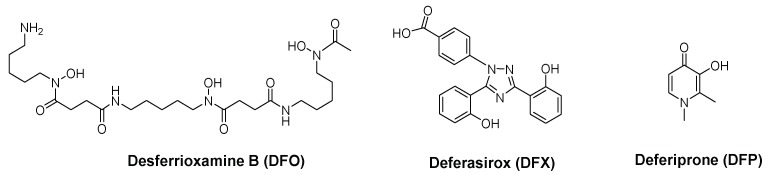
Iron chelators in clinical use: desferrioxamine B (DFO), deferasirox (DFX) and deferiprone (DFP).

Natural and synthetic Fe chelators have first been used to treat iron overload diseases and at present three types of chelators (desferrioxamine (DFO), desferasirox (DFX) and deferiprone (DFP)) (Figure 1) are used in clinical practice [18,20,21,22]. The use of Fe chelators has been extended to address the control of Fe levels in other disease scenarios in which a disruption of Fe homeostasis has been identified such as inflammation, infection, cancer, neurodegenerative processes and diabetes [23,24,25].

Although we recognize the importance of the use of chelation therapeutic approaches in many diseases in this work we will focus on infection, particularly on mycobacterial infection.

### 1.2. Iron and Infection

The sharp control of transition metal ion concentration is of extreme importance in infection scenarios since transition metals are also nutrients for the invading pathogens. As referred above, living organisms counteract transition metal ion toxicity through mechanisms that maintain very tiny amounts of free metal ion available for redox cycling. Similar mechanisms seem to be used by living organisms to withhold nutrients from invading pathogens in a process, which has been termed “nutritional immunity” [26,27].

In fact, it has been described that the withholding of Fe is one of the primary defence mechanisms against pathogenic invaders [26,28]. The competition for Fe between pathogens and their hosts is dramatic and pathogenic bacteria have developed survival strategies like residing in specific intracellular niches within the host cell and the ability to use the host Fe sources. Mycobacteria, namely *M. tuberculosis* and *M. avium*, are examples of intracellular pathogens, being able to inhibit the maturation of phagosomes and taking profit of the host Fe acquisition mechanisms [29].

An increase in the susceptibility to infection was observed in patients suffering from Fe overload related diseases [30]. Due to the high levels of iron available in Haemochromatosis, the disease has been associated with an increased susceptibility to infection by several bacteria [27] such as *Yersinia enterocolitica* [31] and *Vibrio vulnificus* [32]. High levels of iron acquired from the diet lead to an exacerbation of *M. tuberculosis* infection symptoms in both humans and mice [33]. Moreover, it has been shown that iron overloaded haemochromatosis protein (HFE) deficient mice are more susceptible to *M. avium* infection [34]. These findings were reproduced in macrophages isolated from mice and infected with *M. avium* [35]. Several studies correlate the iron status in AIDS patients and the susceptibility to mycobacterial infections and experiments have shown that iron overload increases mycobacterial growth in immune-deficient mice [35,36], and iron redistribution towards macrophages in AIDS patients was shown to favour the progress of tuberculosis [37].

Taking advantage of the knowledge on the iron-related interplay between host and the pathogen and the evidence of a nutritional immunity, the development of many different antibacterial approaches based on the destabilisation of iron concentrations accessible for bacteria have been reported [26,38,39,40].

Amongst others, the process of iron acquisition represents one of the pathways, which can be successfully targeted by new antibacterial drugs and restriction of Fe was shown to improve the outcome of a number of infectious diseases including mycobacterial infections [29,41,42,43,44].

Considering that bacteria produce siderophores to gain access to iron from their environment one of the obvious choices to intervene in Fe acquisition is the administration of synthetic chelators that are able to compete with the natural ones. Preferably, from the chemical point of view the structure of new antimicrobial agents should differ from that of natural siderophores in order to prevent the recognition and uptake of the iron-chelate by the pathogens.

#### 1.2.1. Iron Chelation—A Therapeutic Tool to Tackle Microbial Infection?

Several reports have been produced, suggesting the application of chelation therapy for the control of infections [29,38,45,46,47,48].

The effect of iron chelators in the treatment of bacterial infections has been extensively investigated and one of the first molecules studied was 8-hydroxyquinoline and its related ligands [49]. Some chelators such as EDHPA, EDTA and DTPA have shown activity against Gram-positive and Gram-negative bacteria (reviewed in [50,51]). The effect of iron chelators against bacterial biofilms has also been proved, for instance in the biofilms produced by *Pseudomonas aeruginosa* [52,53,54,55] or *Staphylococcus aureus* [56].

The extensively used DFO and DFX have demonstrated antibacterial effect in a numerous range of bacterial pathogens [57,58,59,60]. However, these ligands offer some drawbacks. DFX is a tridentate ligand and consequently has lower iron affinity than most bacterial siderophores, which are hexadentate ligands. DFO is a hexadentate natural siderophore and some pathogenic bacteria possess specific receptors for capturing the iron loaded chelator and use the metal ion for their own growth [59,61,62].

To overcome these disadvantages, the antimicrobial activity of chelating units, which are not present in natural siderophores, such as 3,4-HPO ligands, has been considered. DFP has shown efficacy against several bacteria, such as *S. aureus* and *P. aeruginosa* [59,62,63,64]. Other 3,4-HPO, have shown in vitro inhibitory activity against several Gram-positive and Gram-negative bacterial species, including *S. aureus*, *P. aeruginosa* and *Escherichia coli* [40,65,66,67,68,69,70].

#### 1.2.2. Mycobacterial Infections

##### Mycobacteria

Mycobacterium is a genus of bacteria that comprises several species, such as *Mycobacterium tuberculosis* and *Mycobacterium leprae*, responsible for tuberculosis and leprosy. Other mycobacteria are responsible for opportunistic infections in humans and other animals, namely *Mycobacterium avium*. *M. avium* complex (MAC) includes *M. avium* and also the non-tuberculous mycobacteria, such as *Mycobacterium intracellulare.*

Mycobacterium species can be classified in different groups, according to their growth rate, as slow growers and fast growers. Both *M. tuberculosis* and *M. avium* are slowly growing mycobacteria while other mycobacteria, such as *M. smegmatis* comprises the fast growers group [71]. Mycobacteria can also be classified according to their morphotypes as smooth or rough, transparent or opaque. The smooth transparent morphotype is usually related with virulence in mouse while the rough opaque morphotypes was found in AIDS patients [72].

**Figure 2:**
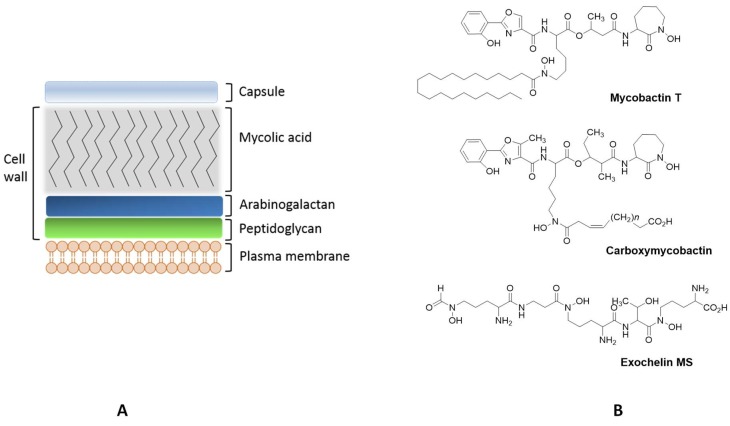
Schematic representation of the cell envelope of pathogenic mycobacteria (**A**, adapted from [74]); Structure of mycobacterial siderophores: Mycobactin T produced by *M. tuberculosis* (MB T); Carboxymycobactin synthetized for *M. avium*, *M. tuberculosis* e *M. bovis* (*n* = 2–9); Exochelin MS produced for *M. smegmatis*. (**B**, adapted from [76,77,78]).

Both, the lipidic composition and the cell envelope architecture contribute to the pathogenicity of these microorganisms [73]. Mycobacteria’s cell envelope is a unique and complex structure composed by the plasma membrane, the cell wall and the capsule (Figure 2) [74].

The lipidic composition of the plasma membrane includes different phospholipids and glycolipids embedded in the plasma membrane, Mycobacteria are unusual Gram-positive bacteria in which the cell wall contains peptidoglycan covalently bound to arabinogalactan esterified with long chain mycolic acids. The more external layer of the mycobacterial cell envelope, the capsule, is predominantly composed by glycoproteins, arabinomannan and mannan derived polysaccharides [74,75]. The particular characteristics of the cell envelope, mainly hydrophobicity and impermeability, contribute to the virulence of mycobacteria, constituting a barrier that compromises the uptake of many antibiotics.

Both *M. tuberculosis* and *M. avium* are facultative intracellular pathogens that reside primarily inside mononuclear phagocytes, namely monocytes and macrophages. These pathogens grow inside vacuoles, the phagosomes, and developed several strategies to survive and resist to host defenses [79,80,81].

To ensure survival and replication inside the phagosome in infected macrophages, mycobacteria developed strategies to retard phagosome maturation, namely by manipulating the host cell endocytic pathways in order to prevent the fusion of the phagosome with late endosomes and lysosomes [79,82]. Thus, the mycobacteria-containing phagosomes remain with characteristics of an early endosome. The levels of ATPase are low and the vacuole is not acidified (the neutral pH value is maintained).

##### Mycobacterial Siderophores

Mycobacteria have developed systems for sensing and regulating iron levels in the intracellular medium. They produce siderophores for iron acquisition and transport and also synthesize proteins to store the iron recruited from the host [83]. Mycobacteria differ from other bacteria by being able to produce different types of iron ligands, namely lipophilic molecules restricted to the cell envelope (mycobactins) and polar extracellular siderophores (carboxymycobactins and exochelins) [83,84] (Figure 2). *M. leprae* is an exception, lacking the gene cluster (*mbt*) responsible for the synthesis o mycobactins [85]. Non-pathogenic species, such as *Mycobacterium smegmatis* and *Mycobacterium neoaurum*, produce exochelins while pathogenic mycobacteria synthesize carboxymycobactins [86,87]. Mycobactins are salicylate-containing siderophores, which are acylated with long chain fatty acids in order to remain associated with the bacterial membrane. The extracellular siderophores are exochelins, which have a peptide-based structure, and/or carboxymycobactins, in which the long alkyl chain of mycobactin is substituted with a short carboxylated acyl chain (Figure 2). Mycobacterial siderophores are hexadentate ligands possessing chelating units based on the phenyloxazolidine ring, ornithine- derived hydroxamates and salycilates [86]. In addition to their specific siderophores, mycobacteria produce salicylic and citric acids which may also play a role in iron acquisition [88].

After production of siderophores and chelation of iron, mycobacteria uptake this element for usage or storage and the mechanism of transport differs according to the type of siderophore and its localization. Mycobactins, located in the membrane, provide high stability constants for the formation of the Fe complex (*Log β* ~ 31) [89] and these siderophores are able to remove iron from host proteins, such as transferrin and ferritin. However, due to their localization, the access to these proteins is difficult sometimes compromising acquisition of such iron sources. Thus, mycobactins bind iron previously bound by carboxymycobactins, which are extracellular siderophores, and then transfer the element through the bacterial membrane [76,87].

Several mechanisms to acquire and transport iron bound to mycobactins have been proposed and in an interesting study Groves et al. [83,90] suggest that mycobactins may diffuse into the host macrophage and then mobilize iron available in the intracellular pools of the macrophage. The ferri-mycobactins are then associated to lipid droplets and return to the phagosomes.

In the case of exochelins, these ligands are able to transport iron through the cell membrane [86,91]. Exochelins, like carboxymycobactins, may also transfer iron to mycobactins. Moreover, it is also described in the literature that exochelins are taken up by active transport and access iron from ferritin, Tf and Lf [76,92].

##### Iron Chelators to Control Mycobacterial Infection

The use of chelators to control mycobacterial infection by iron deprivation has been successfully tested (reviewed in [16,42]). The use of chelators to restrict the iron available for mycobacterial growth has been reported [87], namely for *M. tuberculosis* [43,93] and *M. avium* [42,89,94,95].

DFO has been used to inhibit *Mycobacterium aurum* growth [96] and another study suggested that DFO in combination with silybin, an iron-chelating agent from plants, was able to reduce the extracellular growth of *M. tuberculosis.* However, both of these chelators induced slightly effects on the reduction of in intracellular growth of the pathogen [97], DFO also inhibited biofilm formation by *M. smegmatis* and *M. bovis* BCG [98].

Other ligands, namely spiro pyridopyrrolizines, pyrrolidines [99] and 4*H*-pyrano[3,2-*c*]pyridine derivatives [100], have been tested and the results have shown that the compounds are effective to inhibit the in vitro growth of *M. tuberculosis.* Spiropiperidin-4-ones derivatives also inhibited the growth of *M. tuberculosis*, both in vitro and in vivo [101]. 2-Hydrazinopyrimidin-4(3*H*)-one derivatives, have been tested against *M. tuberculosis* and several molecules have shown antimycobacterial activity [102].

The antimycobacterial activity of different chelators, namely DFO, HBED, dDFT and VUF-8514 against *M. avium* were investigated [42]. The results show that DFO, HBED e VUF8514 inhibit mycobacterial growth in axenic conditions and HBED and DFO also inhibit *M. avium* intramacrophagic. In vivo, the administration of DFO or HBED had small effect on the growth of the pathogen. Considering that inhibition of mycobacterial growth was observed in mice fed with a low iron diet, the authors assume that the limited effect of the chelation therapy is a consequence of a lack of efficacy of currently available chelators, stressing the requirement for new molecules, which can target the intracellular compartments where mycobacteria proliferate [73].

Also, the study of the effect of DFP and other 3,4-HPO, namely L1Net, L1NPr and L1NAll in in vitro studies revealed that the influence of the chelators in the replication of *M. avium* might depend on the tested concentration. Chelators enhanced intracellular and extracellular growth of *M. avium* at low concentrations. However, at high concentrations, they are effective in the control of *M. avium* infections [103].

Functionalized tetrahydro-4(1H)-pyridinones have been synthesized and tested revealing in vitro activity against *M. tuberculosis* [104]. These studies, although demonstrating the effectiveness of an iron deprivation strategy to fight proliferation of mycobacteria also pointed out the necessity to design chelators with physico-chemical properties which allow them to reach the infection targets.

Moreover, at the starting point of our work little was known about the cellular distribution of previously tested chelators thus suggesting the need to synthesize molecules that could be followed inside the cell in order to monitor the chelators pathways inside the cells.

Our research group has designed 3,4-HPO chelators to address *M. avium* infection and performed studies concerning their antimycobacterial activity, interaction with biological membranes and cellular distribution. In this work we discuss the design of chelators, structure activity relationships, combined therapeutic approaches and forthcoming perspectives regarding other bacterial infection scenarios.

## 2. Design of 3-Hydroxy-4-pyridinone Chelators to Address Mycobacterial Infections

### 2.1. Overview

The design of new antibacterial drugs with diverse modes of action is crucial to counteract bacterial resistance [105,106,107]. Within this scope, several studies demonstrated that iron deprivation induced by chelators could be a strategy to inhibit the proliferation of pathogenic bacteria but more effective iron chelators able to reach the infection targets are needed.

We focused our interest in the design of chelators to address mycobacterial infection, in particular infection by *Mycobacterium avium*. Considering the molecular structure of mycobacterial siderophores and bearing in mind that in order to compete for Fe different chelating units, that originate chelators with higher affinity for Fe(III) are necessary, we selected ligands of the 3-hydroxy-4-pyridinone class to achieve our purpose. This class of chelators and their complexes have proved to be useful in several fields of application [95,108,109,110,111]. In what concerns biomedical applications [112,113], Deferiprone (1,2-dimethyl-3-hydroxy-4-pyridinone) is one of the ligands in clinical use to treat iron overload in thalassemia major patients [114,115,116,117].

After choosing the type of chelating unit it was also our objective to design chelators that could be monitored inside the cell and for that reason we designed chelators that could be functionalized with fluorophores. We didn’t realize at the time that the incorporation a fluorophore would be relevant for the antimycobacterial effect or determine the affinity of the chelators towards biological membranes. From a biophysical perspective, this is a fascinating illustration of the fact that functionalization of a molecule with a particular label, to allow spectroscopic detection, may lead to a change in the membrane permeation properties of the molecule and result in a dramatic change in biological activity.

Interesting works calling the attention for the alterations introduced by fluorophore labelling have been reported [118,119,120,121,122,123,124,125] for cell penetrating peptides (CPP) [123]. Two of the works [118,126] regard the antibacterial properties of rhodamine B-conjugated gelsolin-derived peptides and describe the importance of rhodamine B fluorophore on the interaction of the peptides with the bacterial membrane. The authors demonstrate the existence of a positive correlation between the surface pressure activity of the peptides and its antibacterial function, which is based on membrane disruption.

More recently, two elegant studies report the influence of fluorophore labelling on the cellular distribution and cell viability of a set of CPPs [120] and on their interaction with biological membranes [122]. The studies point out the importance of the fluorophore labeling on the alteration of physicochemical properties and demonstrate the existence of correlations between: (i) physicochemical properties and uptake and toxicity of the CPPs [120] and (ii) physicochemical properties and mode and degree of interaction of CPPs with biological membranes [122]. In the latter study it has been found that the most hydrophobic compounds are those that induce the highest membrane disturbance. For the above reasons we believe it is interesting to give a critical account of our entire work in this area and justify the statement “to label means to change”.

### 2.2. 3-Hydroxy-4-pyridinone (3,4-HPO) Chelators

**Figure 3:**
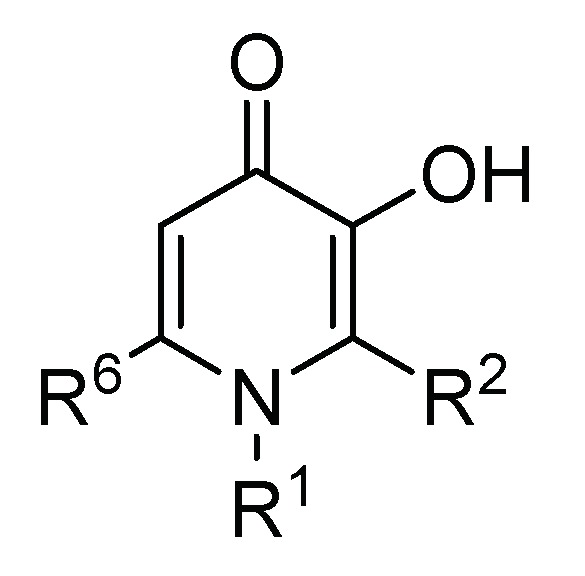
General formula of the 3,4-HPO bidentate chelating unit.

3-hydroxy-4-pyridinones are *N*-heterocyclic compounds in which two adjacent *carbonyl* and *hydroxyl* groups provide the two potential coordinating oxygen atoms that confer the characteristics of a bidentate chelator (Figure 3). The 3,4-HPO ligands display superb chelating properties for M(III) and M(II) metal ions and form M(3,4-HPO)_3_ and M(3,4-HPO)_2_ neutral metal ion chelates. Since the structure of 3,4-HPO ligands allows modification of their hydrophilic/lipophilic balance (HBL) without significantly changing their metal ion affinity, these ligands are particularly suited for biomedical applications. The variation of the HBL of the ligand is also reflected in their corresponding M-chelates.

The versatile chemical structure of 3,4-HPO bidentate chelators has prompted their extensive use as chelating units to synthesize chelators with higher denticity (such as tetra- and hexadentate chelators) [89,95,112,113,127,128].

### 2.3. Anti(myco)bacterial Effect in Intramacrophagic Growth of M. avium

In order to perform a systematic study of 3,4-HPO chelators, and taking into account the results of a previous study regarding the inhibition of intramacrophagic growth of *M. avium* [42], we organized experiments to screen the influence of several properties of the chelators such as denticity, lipophilicity and structure of anchor molecules used to produce hexadentate chelators.

**Figure 4:**
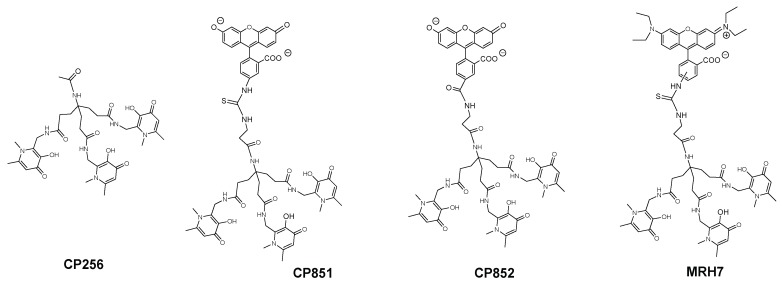
Formulae of 3,4-HPO chelators, CP256 (non-fluorescent ligand), CP851, CP852 (green ligands) and MRH7 (red ligand).

The results showed that, like deferiprone, none of the seven *N*-alkyl and *N*-aryl bidentate chelators was able to inhibit intramacrophagic growth of *M. avium*. Since the chelators tested exhibit variable lipophilicity we ruled out or the influence of the substituents for this first group of chelators [94]. Hexadentate chelators were synthesized using three types of anchor to bind the chelating bidentate units. One of the anchors (CP256, Figure 4) is based on a tetrahedral carbon atom thus providing an extra binding site further functionalization. This possibility was ideal for the synthesis of fluorescent chelators and being so, compounds labelled with rhodamine B (MRH7 in Figure 4, referred as CP777 in reference [94] and as ligand 4 in reference [89]) and fluorescein derivatives (CP851, CP852, Figure 4) were synthesized and tested against *M. avium* intracellular infection of bone marrow-derived macrophages (BMM).

The results showed that the chelating unit CP256 did not affect *M. avium* growth inside BMM but contrastingly the rhodamine B labelled chelator had a clear and reproducible inhibitory effect. In order to confirm that the effect of MRH7 was due to its capacity to chelate Fe we tested a parent chelator in which the chelating units were chemically blocked and the Fe(III) complex of MRH7. Upon addition of the latter compounds we observed no effect in *M. avium* growth. Comparison of the effect of chelators labelled with rhodamine B and fluorescein derivatives showed that, although all of them have a better effect than the non-functionalized chelating unit the effect of MRH7 is much better in particular for lower concentrations [94].

The differences in anti(myco)bacterial effect observed between the hexadentate unit and the rhodamine labelled chelator bearing the same chelating unit, were quite fascinating since both compounds have the same affinity for iron (*Log β* (Fe^3+^) = 34.4 and pFe^3+^ = 29.8), values which are greater than those of natural siderophores. The results demonstrate that a strong capacity to chelate iron is indispensable but that characteristic alone is not sufficient to inhibit *M. avium* growth. Moreover, the differences between chelators labelled with two types of fluorophore also suggest that the structure of the fluorescent group may be significant.

At this stage, we hypothesized that the better anti(myco)bacterial effect of the rhodamine B labelled chelator must also be associated with a special ability to penetrate the cell and to gain access to its targets. In order to get insight on such differences we studied the permeation properties and cellular distribution of chelators MRH7 (rhodamine B labelled) and CP852 (fluorescein labelled) [89]. The values of partition coefficients (*Log* D_7.4_) and of partition constants (*K*_P_) in liposomes showed that MRH7 is strongly lipophilic and has a strong affinity for lipid bilayers in contrast with CP852. The intracellular distribution of the two chelators is markedly different. Upon 20 min of incubation, chelator MRH7 is widely distributed in the cell and accumulates in phagosomes while CP852 is localized in vesicles and areas proximal to the plasma membrane but not inside phagosomes. The results suggest that the structure of the rhodamine labelled chelator seems to be more suited to progress within the cell and reach the niche that harbours *M. avium*.

**Figure 5:**
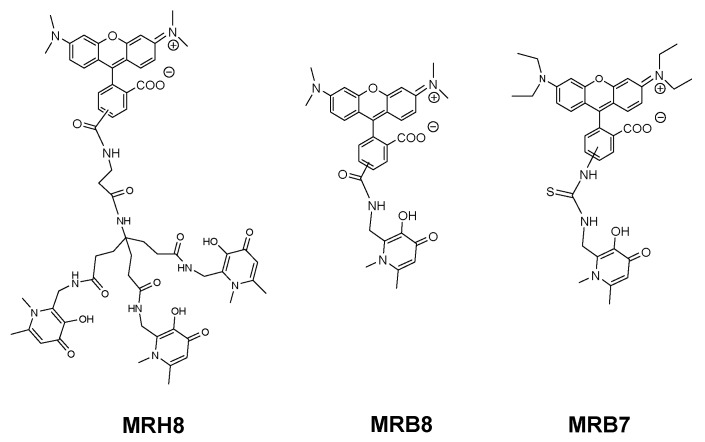
Formulae of fluorescent 3,4-HPO chelators, MRH8, MRB8 and MRB7. The abbreviation and numbering of compounds was assigned according to chelator denticity (MRB*i* for bidentate and MRH*i* for hexadentate) and fluorophore (*i* = 7, 8).

To better understand the influence and consequence of the rhodamine B fragment we designed a hexadentate chelator conjugated with another rhodamine derivative (5(6)-carboxy-tetramethylrhodamine) and the corresponding bidentate chelators labelled with the same fluorophores [95] (Figure 5).

The results of this new experiment showed that: (a) 5(6)-carboxytetramethylrhodamine- labelled chelators are capable to limit intracellular growth of *M. avium*; (b) administration of bidentate fluorescent chelators also restricts intracellular *M. avium* growth as long as the concentration used is corrected according to the stoichiometry of the Fe chelate; (c) the antibacterial effect is dependent on the structure of the fluorescent label and (d) the inhibitory effect of the rhodamine B labelled compounds (MRH7 and MRB7) is significantly superior to that observed for the tetramethylrhodamine labelled chelators (MRH8 and MRB8). This result points out the importance of the rhodamine B fluorophore and the thiourea linkage, which has been introduced in the molecular framework by the use of the reagent rhodamine B isothiocyanate in the synthetic procedure. For chelators MRH8 and MRB8 the rhodamine derivative used is 5(6)-carboxytetramethylrhodamine, which introduces an amide linkage. The type of linkage present in the chelators was not deliberate, but remarkably, the choice of different types of reagents revealed that apart from the fluorophore, the linkage between chelating unit and fluorophore must also be considered in the chelators ´design. We consider the possible relevance of the thiourea group since: (a) the more efficient chelators contain that type of linkage and (b) the antibacterial properties of isothiocyanate groups against Gram(+) and Gram(−) bacteria, which have been related to the capacity of the group to disorder the structure of the bacterial membrane, and are well-known [129,130,131].

We speculate that the presence of the thiourea linkage in the structure of the chelators may allow the targeting of the bacterial cell wall. This effect would not be related with the restriction of Fe sources but might threat the survival of the pathogen. This hypothesis led us to consider the design and testing of other compounds in which the thiourea linkage is now deliberately included in the molecular framework.

**Figure 6:**
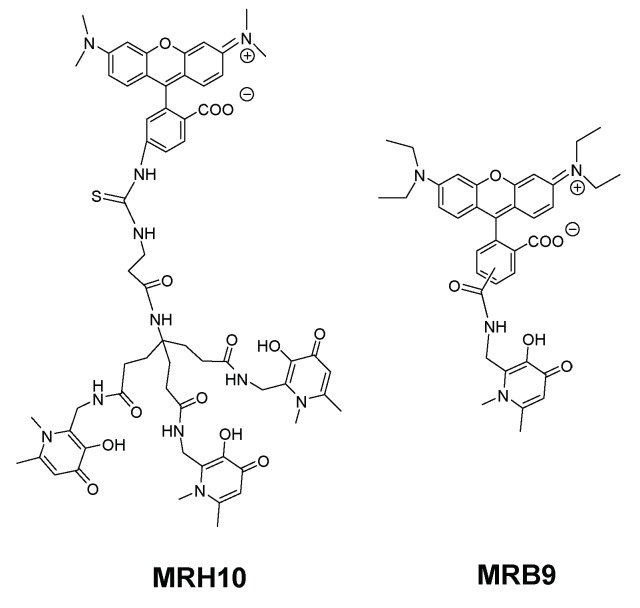
Formulae of fluorescent 3,4-HPO chelators, MRH10 and MRB9. The abbreviation and numbering of compounds was assigned according to chelator denticity (MRB*i* for bidentate and MRH*i* for hexadentate) and fluorophore (*i* = 9–10).

To achieve that purpose we synthesized the set of compounds in Figure 6 and tested their activity against *M. avium* in comparison with that of the lead chelator MRH7 [109]. The results showed that all the fluorescent chelators exhibit antimycobacterial effect and corroborated the relevance of the thiourea linkage, the ethyl substituents on the amino groups of the xanthene ring and the advantage of their associated inclusion in the molecular framework. The rhodamine B labelled chelator MRH7 proved to be the most active compound in controlling of the infection.

In what concerns antimycobacterial infection we think that the next step is the use of combination therapies involving iron chelators and classic antimycobacterial antibiotics in clinical. Such combination allows intervention towards different targets and synergic effects are to be expected. The clinical potential of the combination of iron chelators with other antibiotics has been demonstrated to fight bacterial [39,40,57,58,70], fungal [132] and protozoal [133] infections.

In a first attempt, we investigated the combined administration of chelator MRH7 with the antibiotic ethambutol [109]. Ethambutol, acts by inhibiting the biosynthesis of components of the mycobacterial cell wall [134,135,136]. The compound is active against *M. avium* pathogens but high doses are necessary thus suggesting the application of combined therapeutic approaches. The results showed that the concomitant administration of chelator MRH7 and ethambutol is advantageous since a higher antimycobacterial effect is achieved. The results imply that this combination allows reduction of the amount of chelator used to obtain a significant biological effect and improves the activity of ethambutol.

### 2.4. Chelator Membrane Interactions

In parallel to the antibacterial investigations, biophysical studies regarding the partition and distribution of fluorescein or rhodamine derived chelators have been performed in liposomes membrane models [89,137,138,139].

Drug-membrane interactions have enormous importance in drug design and the understanding of these interactions at a molecular level allows the development of more effective drugs [140,141,142]. In the context of *M. avium* infection, the ability of drugs to interact with and cross barriers is particularly important since *M. avium* is a facultative intracellular pathogen that resides within mammalian cells.

In our studies drug membrane interactions were assessed in liposome model membranes by fluorescence spectroscopy and nuclear magnetic resonance (NMR) and by computational studies, namely molecular dynamic (MD) simulations.

Steady-state fluorescence spectroscopy was used to determine partition constants (*K*_P_) of chelators MRH7, MRB7, MRH8 and MRB8 in DMPC (1,2-dimyristoyl-sn-glycero-3-phosphocholine) and DMPG (1,2-dimyristoyl-sn-glycero-3-phospho-(1′-rac-glycerol)) liposomes [89,137].

Although we recognize that octanol-water *Log* P and *Log* D values give important information regarding the hydrophilic/lipophilic balance of a candidate drug, we believe that the values of partitions constants determined in liposome membrane models are more realistic. The values of *K*_P_ allow a quantification of the interaction of compounds with a lipid bilayer and reflect their preference for the lipid or the aqueous phase. Preparation of liposomes, with lipids of different characteristics allow a better understanding of surface interactions and permeation properties. The values of *Log K*_P_ obtained for chelators with different fluorophores and linkages clearly indicate that compounds incorporating *N*-ethyl substituents in the amino groups of the xanthene ring and a thiourea linkage, have a greater affinity for the lipid phase (*ca* 10 times) than those bearing *N*-methyl substituents and an amide linkage. The results demonstrate that the permeation and partition properties of rhodamine labelled chelators can be tuned by choosing appropriate substituents of the rhodamine moiety.

NMR spectroscopy was also used to determine the affinity of MRB7 and MBR8 for the phospholipid bilayer of DMPC liposomes in order to complement/corroborate the fluorescence studies. The permeation properties of the chelators were assessed by analysis of the alterations of NMR parameters, such as chemical shifts, line shape, spin−lattice relaxation time (T1), and translational diffusion coefficient of the lipids and the liposomes [138,139].

In a first study [138] we used a concentration of chelators within the range of those used in the biological experiments and observed changes in the chemical shifts of the protons associated with the different functional groups of the phospholipid. The latter can be related with a different distribution and location of MRB7 and MRB8 in the phospholipid bilayer of DMPC. The changes in the chemical shift values imply that chelator MRB7 strongly interacts with the choline head groups at the surface of the liposome sphere and is able to permeate deeper and reach the centre of hydrophobic area of the phospholipid bilayer as demonstrated by the significant perturbations of the proton resonances induced on the terminal protons of the acyl chains located in that area. In contrast, MRB8 molecules strongly interact with the polar surface of liposomes and seem to be preferably located between the polar interface and ester groups of the lipid bilayer thus justifying the non-perturbation of the proton resonances belonging to the lipid acyl chains. The presence of *N*-ethyl groups in the xanthene structure and the thiourea link in the structure of MRB7, as opposed to *N*-methyl groups in the xanthene structure and an amide link in the structure of MRB8, seem to facilitate the affinity of MRB7 molecules to the liposome surface and their ability to penetrate deeper into the hydrophobic interior of lipid bilayer.

A more extensive and detailed NMR analysis was performed using higher concentrations of chelators to allow generation of NMR signals of appropriate intensity for measurements based on the NMR resonance signals of the chelators [139]. This study revealed that these chelators, in particular MRB7, might be able to induce alterations in the structure of the liposome. This result is quite relevant since it is indicative that these chelators may be able to disturb the structure of the biological membranes.

MD simulation studies demonstrated that chelators interact with the lipid phases at different levels of the bilayer and that the interaction seems to be reinforced for the compounds that contain *N*-ethyl groups and a thiourea linkage (MRB7 and MRH7). The rhodamine B labelled chelator MRB7 seems to have a superior insertion and residence time in the hydrophobic region of the membrane bilayer, in comparison to the tetramethylrhodamine labelled chelator MRB8. This observation is consistent with the partition constants determined by fluorescence spectroscopy and mainly with NMR results. Altogether, these results support the hypothesis that the effectiveness of the chelators as anti(myco)bacterials is related to a greater ability to permeate lipid bilayers [95,138,139].

All the biophysical results are indicative that the more effective chelators are those that exhibit higher affinity towards lipid bilayers and better permeation properties across biological membranes. These results are in agreement with those obtained for cell penetrating peptides [118,119,120,121,122,123,124,125,126] thus reinforcing the idea that the presence of groups that enhance the partition and permeation properties within lipid bilayers is relevant in the design of new antibiotics.

### 2.5. Intracellular Distribution and Co-Localization Studies of Rhodamine Labelled Chelators in Macrophages

**Figure 7:**
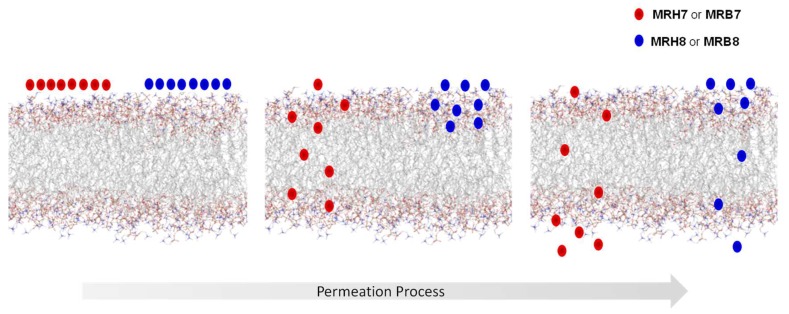
Scheme of a assumed mode of interaction and progress of the rhodamine labelled 3,4-HPO chelators, MRH7 and MRB7 (red) and MRH8 and MRB8 (blue) through the biological membranes, along the permeation process.

Comparison of the intracellular distribution patterns of a rhodamine B labelled chelator (MRH7) and a fluorescein labelled chelator (CP851) in macrophages suggested that distinct uptake and intracellular distributions are likely to account for different efficacy as anti(myco)bacterial agents. To gain insight on the uptake by macrophages and cellular distribution of the set of rhodamine labelled chelators under study we organized new confocal microscopy studies. The results obtained showed that: (i) all the rhodamine labelled chelators are taken up by macrophages; (ii) experiments performed with the same concentration of chelator provide images that suggest that the lipophilicity of the chelators may limit the amount of chelator which is internalized in the cell; (iii) the intracellular distribution and the interaction of the chelators with different cellular compartments within macrophages are similar for all tested chelators; (iv) the rhodamine labelled chelators are able to target the phagosome compartment (v) considering the similarity of the chelators’ distribution patterns in the macrophage and its subcellular compartments, the pathways of all chelators appear to be the same although a considerably higher number of rhodamine B isothiocyanate derived chelators seems to cross the membrane in the same time frame as illustrated in Figure 7.

The concurrent incorporation of *N-ethyl* substituents on the amino groups of the xanthene ring of rhodamine and of a thiourea linkage between chelating unit and the fluorophore produces chelators with enhanced permeation properties and which are superiorly up taken by macrophages. The latter chelators proved to be the most efficient in inhibiting the intramacrophagic growth of *M. avium*.

### 2.6. Suggested Mechanism in M. avium Infection

**Figure 8:**
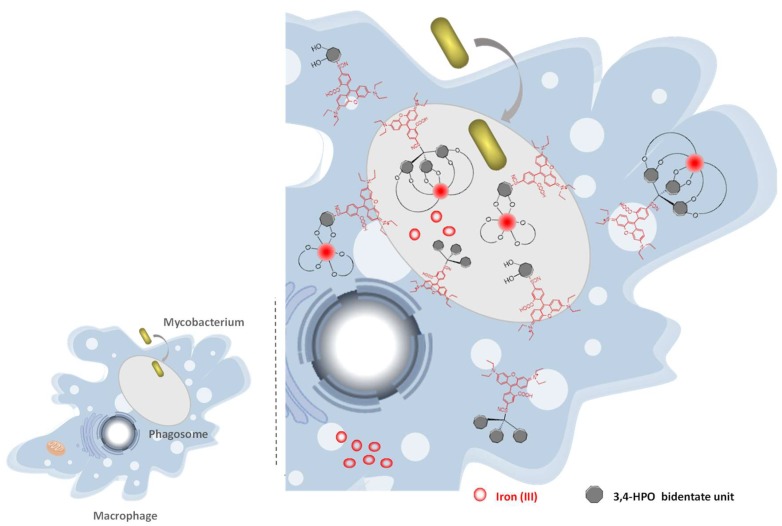
Outline of a hypothetical mechanism for the ironing-out effect produced by the rhodamine labelled 3,4-HPO chelators.

The confocal microscopy studies are indicative that the rhodamine labelled chelators are able to target the phagosome compartment and are likely to preferentially reside in lipid environments. This result is quite relevant since the targeting of the phagosomal compartment was one of our goals. Since we realized that the presence of the fluorophores on the chelators molecular framework is crucial to target the phagosome we hypothesize that the role of rhodamine fluorophore is: (a) to enhance the chelators uptake by the macrophage; (b) to allow access to the phagosome and (c) to efficiently anchor the chelator in the outer and inner parts of the phagosomal membrane thus allowing an efficient competition with natural siderophores and restrict the iron supplies compromising the survival of bacteria (Figure 8). Considering this assumed role of the chelator fluorescent moiety, the better results obtained for the rhodamine B isothiocyanate labelled chelators can be assigned to their higher hydrophobicity and affinity for lipid bilayers.

### 2.7. First Studies in a Different Infection Scenario

As previously referred there is an urgent need to develop new antibiotics that may act on different targets to counteract bacterial resistance. Within this scope, we believe that the use of chelators that are able to deprive bacteria from Fe, can be a choice since the antibiotics currently in use do not target Fe metabolism.

Considering the results obtained in *M. avium* infection, we first investigated the activity of the same set of rhodamine labelled 3,4-HPO chelators towards a selected set of Gram (+/−) bacteria [143]. The results obtained showed that the activity of the fluorescent chelators in this new scenario is quite distinct from the observed in *M. avium*. We found that in Gram (+/−) bacteria only hexadentate chelators have a significant effect.

Regarding the results obtained with the hexadentate chelators we found that MRH7, MRH8, and MRH10 inhibit bacterial growth of *Staphylococcus (S). aureus* ATCC 25923 and *S. epidermis* ATCC 12228. The hexadentate chelator MRH7 is the only one that is able to inhibit the growth of *Escherichia (E.) coli* ATCC 25922. The relevance of the type of fluorophore that provides the thiourea linkage and *N*-ethyl substituents on the xanthene ring was confirmed. Curiously, MRH7 was the only chelator able to have an effect towards Gram (−). Chelator MRH7 exhibits the higher partition constant in liposomes and proved to be able to permeate lipid bilayers [137].

At this point it is interesting to bear in mind the different complexity and composition of Gram (+) or Gram (−) bacterial cell walls. Gram (−) bacteria may be less accessible for the penetration of the chelators and the superior permeation properties like of MRH7 may be relevant to understand the result. However, further studies are necessary to establish structure-activity relationships.

**Figure 9:**
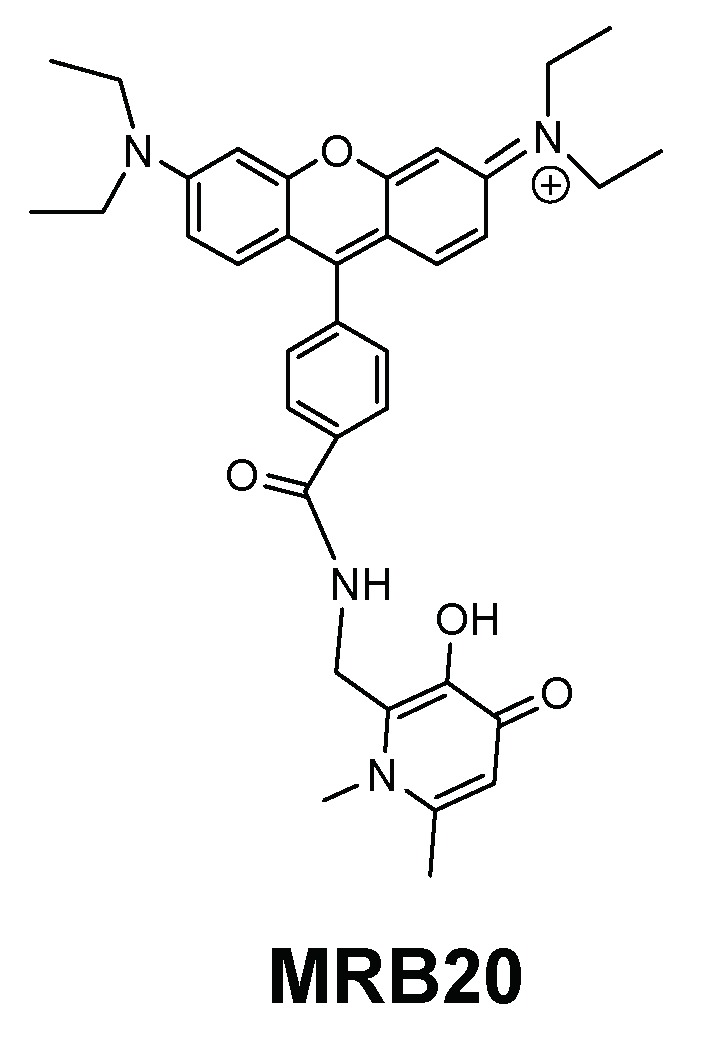
Formula of rosamine-derived 3,4-HPO chelator, MRB20.

For the same type of bacteria, we also investigated the activity of a new 3,4-HPO bidentate chelator labelled with a rosamine xanthene fluorophore (MRB20, Figure 9) in order to evaluate the effect of the chelators´ charge [144]. The rosamine fluorophore is structurally related with the previously described rhodamine moieties but lacks one carboxylic acid substituent thus implying that the overall charge of the chelator is positive, at pH = 7.4, in contrast with the neutral charge of rhodamine labelled chelators. Chelator MRB20 revealed a promising antibacterial activity against Gram (+) strains including clinically relevant species as *S. aureus*, *S. epidermidis*, *Enterococcus (E.) faecium* and *E. faecalis* [144].

## 3. Concluding Remarks and Future Perspectives

The challenge that gave rise to the work described was “*to design Fe chelators to ironing out mycobacteria*”. We consider that the objective was achieved and the work gave a significant contribution to produce chelators with adequate properties for macrophage uptake and distribution inside the cell.

The choice of the class of ligands seems to be adequate since the chelating units are able to compete with mycobacterial siderophores. Although determinant, this chelating capacity is not sufficient to achieve anti(myco)bacterial effect.

The labelling of the chelating unit with xanthene fluorophores proved to be crucial for the anti(myco)bacterial activity. Moreover, we found that the type of linkage that binds fluorophores to the chelating unit also seems to have an effect in anti(myco)bacterial activity . Fluorescein labelled chelators proved to be much less effective than the rhodamine parent compounds. For the latter group we found that the anti(myco)bacterial effect is dependent on the substituents of the xanthene ring groups. For rhodamine labelled chelators the simultaneous presence of *N*-ethyl substituents on the amino groups of the xanthene ring and a thiourea linkage between chelating unit and the fluorophore, produces chelators with enhanced anti(myco)bacterial effect. The same chelators also have the better permeation properties across biological membranes and are superiorly up taken by macrophages. These results point out the significance of the conjugation of a rhodamine B label to the chelator through a thiourea link and suggest that the high affinity of rhodamine B towards lipid phases may be determinant to situate the chelator in a favourable position to successfully compete with mycobacterial siderophores.

We conclude that the effectiveness of the chelators to inhibit the intramacrophagic growth of *M. avium* is strongly dependant on its capacity to interact with and reside in the lipid bilayers. This capacity seems to provide better conditions to deprive mycobacteria from Fe. Confocal microscopy studies proved that rhodamine labelled chelators have access and are able to remain in to the phagosomal compartment, thus corroborating our hypothesis.

It may be relevant to refer that, even though we performed confocal microscopy studies with genetically modified green mycobacteria we never observed co-localization of the red chelator MRH7 and the bacteria. The mode of action may not include the crossing of the bacterial cell-wall but its function may be just ironing out the phagosome or create a gradient between the various macrophage iron pools. However, as the fluorescence of MRH7 is quenched upon chelation of Fe we cannot exclude the possibility that the chelator reaches *M. avium* where it resides as a non-fluorescent Fe-chelate, which we are not able to detect.

In order to complement the studies described herein and regarding *M. avium* infection, we consider that we need to investigate the toxicity of these chelators in several conditions and also to test the chelators in in vivo models. The first results obtained in Gram (+/−) infection scenarios encourage the pursuit of further studies and point out the need to explore the variety of functional groups and charge of the new molecules, as discussed in Section 2.7.

In the studied systems, it was found that chelators that presented a higher capacity to interact with biological membranes also lead to an increased antibacterial effect. In Table 1 we summarize all results regarding the antibacterial effect and partition properties in biological membranes obtained with this set of chelators together with their structural features.

Our results corroborate the findings of other authors [120,122] regarding the modifications induced by the introduction of fluorescent labels in the physico-chemical, biophysical and biological properties of a molecule. Moreover, the results also point out that the choice of reagent derivatives used in the synthesis of the fluorescent molecules may introduce functional groups that further enrich the molecule. The results reviewed herein illustrate the consequence of small changes in the structure of the biological relevant molecules and the influence that these modifications might have in drug- membrane interactions and biological activity.

Considering our results in conjunction with others described in the literature, namely those related with rhodamine derived liposome tags [146], mitochondrial probes [147] and antimicrobial peptides [118,120,122,123,126], some other topics can be further explored.

**Figure 10:**
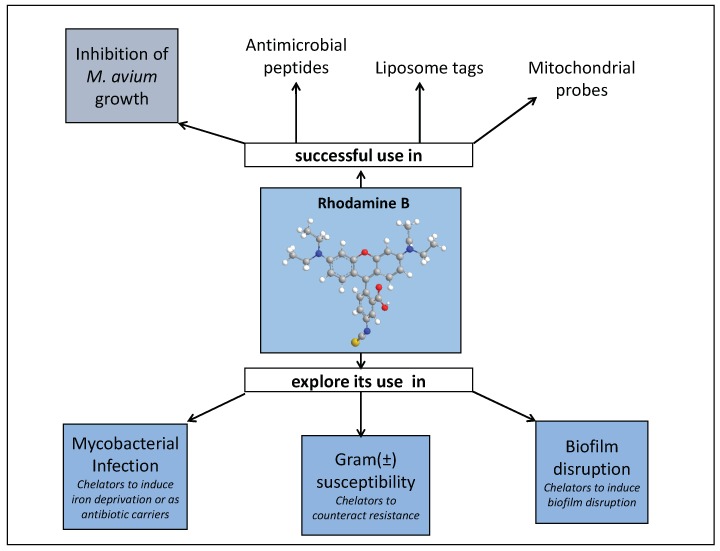
A graphical representation of past and future fields of application of rhodamine B conjugates.

We believe it is worth to investigate the use of rhodamine B conjugates to target mycobacterial infections and closely related problems such as bacterial resistance and biofilm production. Rhodamine B has the advantage of being a fluorescent molecule whose properties are sensitive to its environment thus allowing following its pathway within the cell. Also, it is well known that the formation of biofilms is associated with a hydrated extracellular matrix composed by polysaccharides, nuclei acids, lipids and metal ions. We think that the use of 3,4-HPO chelators could have an effect in the disruption of biofilms produced by several bacterial strains and insight in this area are meaningful. A graphical representation of past and future fields of application of rhodamine B conjugates is depicted in Figure 10.

## Figures and Tables

**Table 1:** Summary of the antibacterial activity, structural features and membrane interaction of the fluorescent chelators. (0–no effect; +–low; ++ moderate; +++–high; ++++ very high effect; *nd*–not determined).

Chelator	Structural Features			Antibacterial Activity		Membrane Interaction	Ref.
	Type of Fluorophore	Linker	Charge (at pH = 7.4)	*M. avium*	*Gram (+/−)*		
MRB7	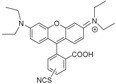	Thiourea	Neutral	++++	0	++	a
MRH7		Thiourea	Neutral	++++	+++	+++	
MRB8	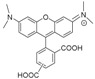	Amide	Neutral	+	0	+	
MRH8		Amide	Neutral	+	+	+	
MRB9	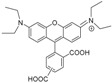	Amide	Neutral	++	0	*nd*	b
MRH10	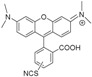	Thiourea	Neutral	+++	++	*nd*	
MRB20	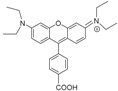	Amide	Positive	*0*	++++	*nd*	c

(a) [95,137,138,139,143,144]; (b) [109,143]; (c) [144,145].
